# Use of RNAi technology to develop a PRSV-resistant transgenic papaya

**DOI:** 10.1038/s41598-017-13049-0

**Published:** 2017-10-03

**Authors:** Ruizong Jia, Hui Zhao, Jing Huang, Hua Kong, Yuliang Zhang, Jingyuan Guo, Qixing Huang, Yunling Guo, Qing Wei, Jiao Zuo, Yun J. Zhu, Ming Peng, Anping Guo

**Affiliations:** 1 Institute of Tropical Bioscience and Biotechnology, Chinese Academy of Tropical Agriculture Sciences, 571101 Haikou, Hainan China; 2 Hawaii Agriculture Research Center, 96797 Waipahu, HI USA; 3 School of Basic and Life Science, Hainan Medical University, Haikou, 571199 Hainan China; 4 Institute of Banana and Plantain, Haikou Substation, Chinese Academy of Tropical Agriculture Sciences, 570102 Haikou, Hainan China

## Abstract

Papaya ringspot virus (PRSV) seriously limits papaya (*Carica papaya* L.) production in tropical and subtropical areas throughout the world. Coat protein (CP)- transgenic papaya lines resistant to PRSV isolates in the sequence-homology-dependent manner have been developed in the U.S.A. and Taiwan. A previous investigation revealed that genetic divergence among Hainan isolates of PRSV has allowed the virus to overcome the CP-mediated transgenic resistance. In this study, we designed a comprehensive RNAi strategy targeting the conserved domain of the PRSV CP gene to develop a broader-spectrum transgenic resistance to the Hainan PRSV isolates. We used an optimized particle-bombardment transformation system to produce RNAi-CP-transgenic papaya lines. Southern blot analysis and Droplet Digital PCR revealed that line 474 contained a single transgene insert. Challenging this line with different viruses (PRSV I, II and III subgroup) under greenhouse conditions validated the transgenic resistance of line 474 to the Hainan isolates. Northern blot analysis detected the siRNAs products in virus-free transgenic papaya tissue culture seedlings. The siRNAs also accumulated in PRSV infected transgenic papaya lines. Our results indicated that this transgenic papaya line has a useful application against PRSV in the major growing area of Hainan, China.

## Introduction

Papaya (*Carica papaya* L.) is a widely cultivated fruit crop in the tropics and sub-tropics. However, the production of papaya is seriously limited by papaya ringspot disease caused by *Papaya ringspot virus* (PRSV) worldwide. Infected papaya plants exhibit symptoms of yellowing, distortion, severe leaf mosaic, and the classic “ringspot” on fruit^1^. Viral infection impacts papaya growth, reduces fruit quality, and prevents fruit set^2^. It is in the genus *Potyvirus* and contains a monopartite, single-stranded, positive-sense RNA^3^. PRSV isolates are grouped into the papaya-infecting type (PRSV-P) that infects both papaya and cucurbits, and the cucurbit-infecting type (PRSV-W) that infects cucurbits but not papaya^2,4^. PRSV is mainly transmitted by aphids in a non-persistent manner^5^.

Papaya and *Arabidopsis thaliana* are members of the *Brassicales*. Genome sequence analysis revealed that the papaya has significantly fewer disease resistance genes than that of *Arabidopsis*. The papaya genome is significantly larger, at 372 mega base pairs (Mbp) vs. 145 Mbp^6^. However, there are fewer nucleotide-binding site (NBS)-containing R genes in papaya than in *Arabidopsis*, 54 vs. 174, respectively^7^. The natural source of lacking effective resistance makes the conventional breeding for resistance difficult^7^. Several methods are used to control papaya ringspot disease, including quarantine, geographic displacement, roguing, netting, cross-protection, and genetic modification of the host plant^8^. Because PRSV is rapidly and efficiently transmitted by aphids, the use of insecticides is impractical^2^. In Hawaii and Taiwan, a mild strain of PRSV, HA5-1, has been used to protect papaya plants against the infection by virulent strains of the virus^9^.

The first commercialized transgenic papaya carrying the PRSV CP gene was introduced to Hawaii in 1998 and saved the remains of the papaya industry^10^. However, CP-transgenic resistance of papaya is expressed in a nucleotide-sequence-homology-dependent manner^11^. Transgenic papaya cultivars have varying levels of resistance against PRSV isolates from other geographical regions. For example, isolates from the Bahamas, Florida and Mexico have delayed, mild symptoms. Isolates from Brazil and Thailand also have delayed symptoms, but the virus eventually overcomes their resistance^12^. The CP hemizygous line, ‘Rainbow,’ is also susceptible to PRSV isolates from Taiwan^13^. Resistance levels therefore depend on the variability among CP genes of the isolates^8,14,15^. We have previously reported that PRSV isolates from Hainan province, China, are highly variable^16^. The high levels of genetic divergence in PRSV isolates from Hainan is likely to be the cause of the failure of transgenic papaya lines that targets specific viral CP genes^16^.

The RNA-mediated resistance in papaya is based on post-transcriptional gene silencing (PTGS), a host defense response to foreign RNA^11^. PTGS-mediated transgenic resistance depends on the sequence homology between a transgene and the corresponding viral genome^17^. Transgenic papaya has been proven to have effective resistance to PRSV isolates from Hawaii, Taiwan, and other. In this work, we use the RNAi strategy to construct a transgene that targets the conserved region of the PRSV CP genes and confers broad-spectrum resistance to the diverse Hainan PRSV isolates.

## Results

### Vector construction and plant transformation

**Figure 1:**
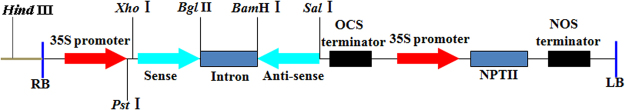
Components of the constructed RNAi vector pCAMBIA2300-35S-OCS. 35 S promoter indicated *Cauliflower mosaic virus* (CaMV) promoter. NOS terminator = nopaline synthase gene terminator, OCS terminator = octopine synthase terminator. NPTII = neomycin phosphotransferase gene. Sense and Anti-sense = conserved 544 bp fragment of CP gene and inverted repeat sequence.

The genetic variability among PRSV isolates in Hainan was investigated in a previous study^16^. Sequence analysis of the 544-bp region of the Hainan PRSV isolates shared 97 to 100% identity (Supplementary Figure 1) were used to design RNAi hairpin structures. The constructed CP hairpin structure was inserted into pCAMBIA2300-35S-OCS to construct the plant expression RNAi vector (Fig. 1). The hairpin structure was validated by digestions with Sal I and Pst I (Supplementary Figure 2).

### Identification of transgenic lines by PCR

**Figure 2:**
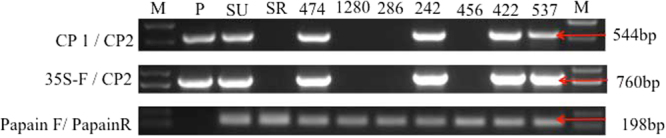
PCR validation of the transgenic event in difference lines. CP1/CP2 primers amplified the 544-bp conserved region of the CP gene in sense orientation. 35S-F/CP2 primers were vector-specific, amplifying the 35 S promoter and conserved sense region of the CP gene. Papain F/ Papain R primers amplified the papaya reference gene. M = DNA molecular weight, P = plasmid DNA, SU = ‘SunUp’ DNA, SR = ‘SunRise’ DNA before transformation. The other numbers are candidate transgene lines. Arrows on the right panel indicated the expected PCR products. The full-length gels are presented in Supplementary Figure 3.

All plants regenerated from embryogenic callus under antibiotic selection were screened for transgenic events by PCR (Fig. 2). Primers amplifying a 760-bp fragment containing the 35 S promoter elements and complete CP hairpin region were used to verify presence of the target transgene (Table 1 and Fig. 2). Regenerated lines 242, 422, 474, and 537 were confirmed to be transgenic by PCR, while line 1280 was not transgenic.

**Table 1:** Primers used in this work.

Target gene	Primer name	Primer sequence (5′-3′)	Size (bp)
CP hairpin sense strand	CP-RNAi-P1	CCCTCGAGTCAACGCCGGAACTAGTGGAACTT Xho I	560
	CP-RNAi-P2	GAAGATCTYCACGAGCCCTATCAGGTGTCTTT Bgl II	
CP hairpin reverse strand	CP-RNAi-P3	GCGTCGACTCAACGCCGGAACTAGTGGAACTT Sal I	560
	CP-RNAi-P4	CGGGATCCTCACGAGCCCTATCAGGTGTCTTT BamH I	
35 S + Coat protein	35S-F	GCTCCTACAAATGCCATCATTGC	760
	CP2	TCACGAGCCCTATCAGGTGTCTTT	
Coat protein	CP1	TCAACGCCGGAACTAGTGGAACTT	544
	CP2	TCACGAGCCCTATCAGGTGTCTTT	
Papain	PapainF	GGGCATTCTCAGCTGTTGTA	198
	PapainR	CGACAATAACGTTGCACTCC	
Helper component proteinases (HC-Pro)	HCProF	TGAATGCACGTAACATGAACGA	484
	HCProR	ACCATTTGCTGCCGAAACCTCT	
Insertion specific	474-61 F	TATAACCCCGCAGGCAATCC	192
	474-61 R	AACTCTGTGGTCCTAAGTGGT	
siRNA probes*	CP544-10	atggaatcaa gaggaatttg actgacatta gcctcgctag atatgctttc	50
	CP544-10-10	gaaagcatat ctagcgaggc taatgtcagt caaattcctc ttgattccat	
	CP544-11	gatttctatg aggtgaactc aaaaactcct gatagggctc gtga	44
	CP544-11-11	tcacgagccc tatcaggagt ttttgagttc acctcataga aatc	

^*^siRNA probes were designed and synthesized to hybridize to the CP gene (sense strand) sequence in 50-bp steps. For example: CP544-1 is from 1 bp to 50 bp, CP544-2 is from 51 bp to 100 bp, … CP544-10 is from 451 bp to 500 bp and its reverse complimentary sequence is CP544-10-10; CP544-11 is from 501 bp to 544 bp and its reverse complimentary sequence is CP544-11-11. The latter two probes (CP544-10/CP544-10-10 and CP544-11/CP544-11-11) have efficient hybridization signal.

### Southern blot analysis to confirm single insert in transgenic line

**Figure 3:**
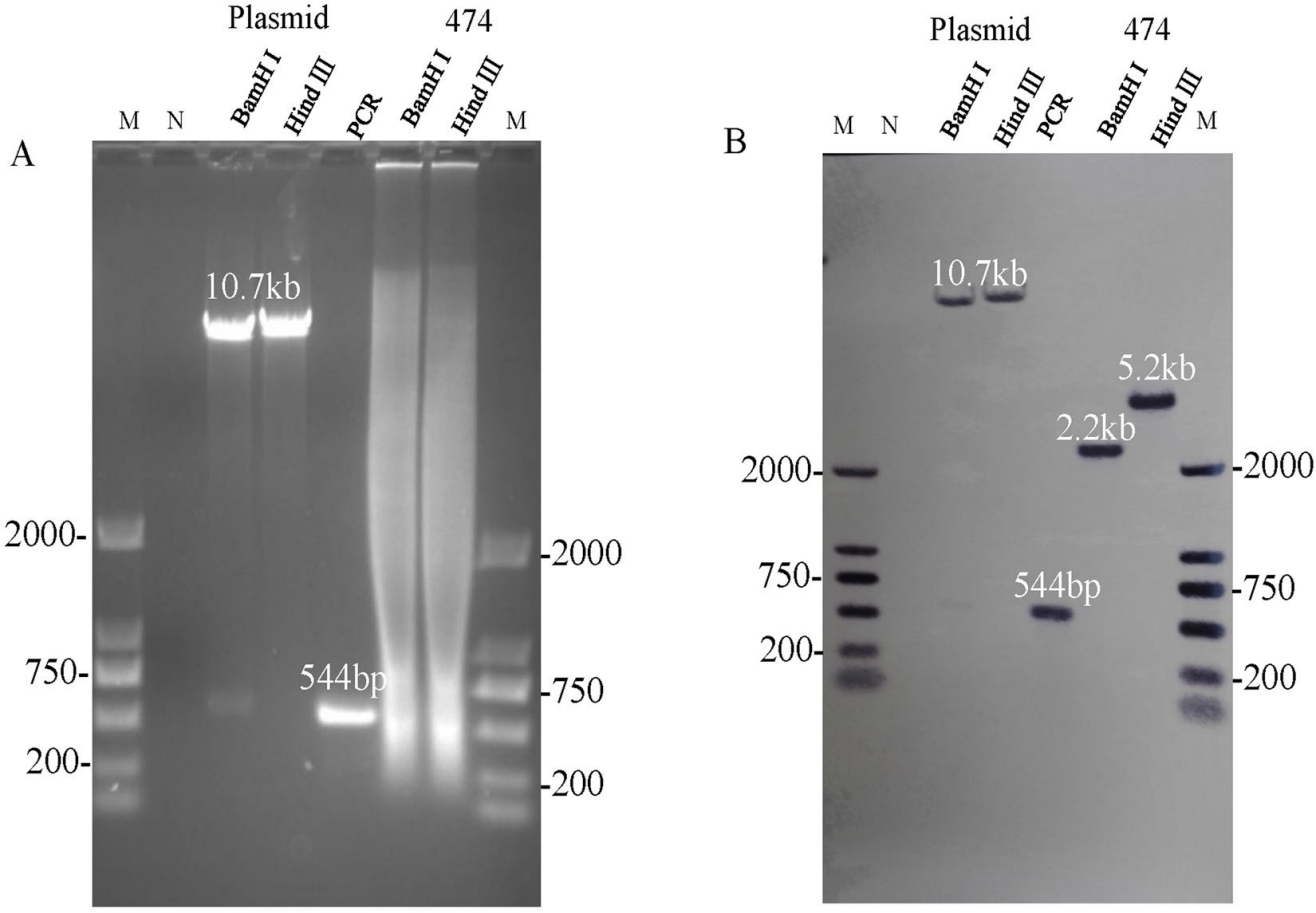
Southern blot analysis confirmed a single insertion site in transgenic line 474. Plasmid and line 474 were digested with BamH I and Hind III separately, and the 544-bp CP gene conserved region was produced by PCR as a reference control. (**A**) Agarose gel image before transfer to nylon membrane. (**B**) Southern blot. The probe of CP fragment and DNA marker were labeled with Digoxigenin-11-dUTP individually, and mixed together before hybridization.

Digestion of line 474 genomic DNA with either Hind III or BamH I produced a Southern blot that showed a single band in each digest, indicating that there is only one insertion site in transgenic line 474 (Fig. 3).

### Using digital PCR to determine copy number in the transgenic lines

Analysis by ddPCR showed that the transgenic insertion in papaya line 474 contained 0.83 copy (about 1) of the CP gene. SunUp is homozygous for the CP gene at a single insertion site (CP/CP)^18^ and therefore has 2 copies of the transgene (Supplementary Figure 4).

### *hi*TAIL-﻿﻿PCR used to locate the insertion site

**Figure 4:**
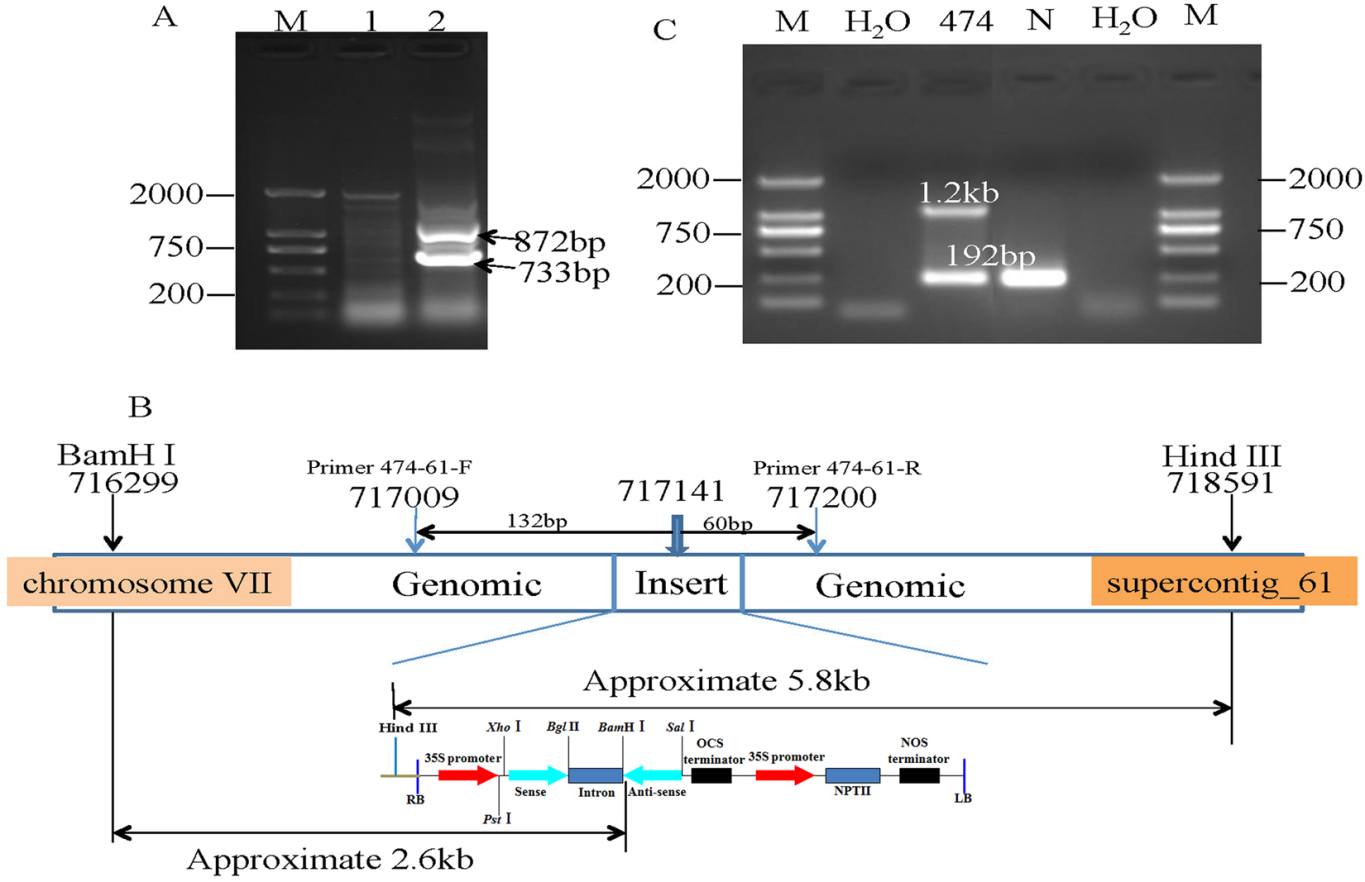
High-efficiency thermal asymmetric interlaced PCR (*hi*TAIL-PCR) verified the insertion site. (**A**) Two rounds of *hi*TAIL-PCR for line 474. M = molecular marker; lane 1 = first round PCR; lane 2 = second round PCR; arrow indicates two products that were subsequently sequenced. (**B**) Structural diagram of the specific insertion site located in chromosome VII, supercontig 61 at 717141 bp. Primer 474-61 F/R were designed to amplify 192 bp in non-transgenic line. (**C**) Nested PCR with primers 474-61 F/61 R bracketing the insertion site and primers CP1/CP2 used to verify the insertion of the CP gene. Line 474 samples amplified as a PCR template produced two bands; one band (192 bp) was the same as the negative control for the insertion event, while the other band was about 1.2 kb.

The genomic location of the insert in transgenic line 474 was determined by hiTAIL-PCR (Fig. 4A). The result showed the insertion site was anchored on papaya chromosome VII, within supercontig_61 at base pair 717,141 (*Carica papaya* ASGPB v4.0, https://phytozome.jgi.doe.gov). The insertion did not lie within any predicted open reading frames (ORFs) (Fig. 4B).

### Transformation-site-specific PCR to confirm the insertion

To further verify the authenticity of the insert, two primers, 474–61 F/474-61 R, were designed to amplify a 192-bp product containing and centered approximately on the insertion site (Fig. 4C). The site-specific primers produced one band of the expected size (192 bp), when amplifying genomic DNA of a non-transgenic control line, but no band was produced when genomic DNA of line 474 was used as template, due to the large size of the inserted sequence. Furthermore, nested PCR containing both a site-specific primer and CP reverse primers was designed to confirm the insertion site. PCR products from line 474 contain a 1.2-kb band (CP inserts + 35 S promoter + plasmid right border + plant genome right wing). Additionally, we also confirmed that line 474 was hemizygous (CP/-), since the nested primers produced both the 1.2-kb site-specific band and the 192-bp band of the unaltered genome (Fig. 4C).

### Bioassays for resistance to Hainan PRSV isolates in transgenic line 474

**Figure 5:**
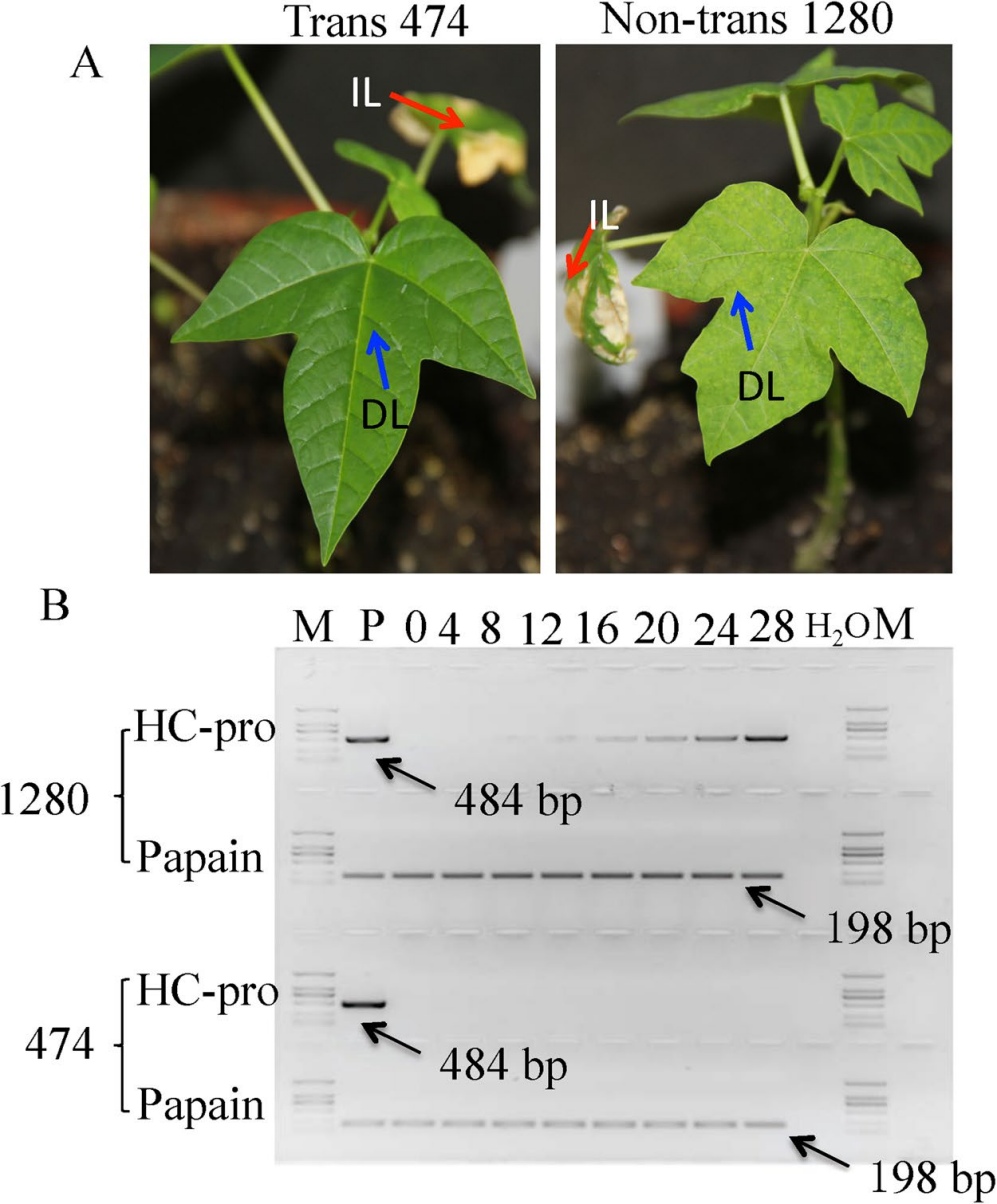
Bioassays of transgenic line 474 to determine resistance response to inoculation with a mixture of PRSV isolates F61d, F10d and F21d. (**A**) Plant inoculation bioassay. Typical PRSV symptoms were observed in non-transgenic line 1280 at 24 DAIs, while transgenic line 474 had no visual symptoms. IL indicates the inoculated leaf, DL indicates the detected leaf. (**B**) Reverse-transcription PCR of the HC-Pro gene (484 bp) was used to detect PRSV. Papain was used as internal reference gene (198 bp). PRSV accumulation in the transgenic and non-transgenic papaya was monitored 0, 4, 8, 12, 16, 20, 24, and 28 days after inoculation. P = plasmid as positive control. H_2_O = nuclease-free water as negative control. M = DNA molecular marker.

Transgenic line 474 and a non-transgenic line 1280 were challenged with severe PRSV isolates of Hainan subgroups I, II and III, as well as a mixture of all three. The accumulation of viruses in inoculated plants was monitored every 4 days from 0 to 28 days after inoculation (DAI) (Fig. 5). Typical PRSV symptoms were observed in line 1280 at 12 DAI, and severe symptoms were observed at 24 DAI. In contrast, line 474 had no visual symptoms (Fig. 5A). Reverse transcription PCR (RT-PCR) using primers specific for the HC-Pro gene of PRSV was employed to semi-quantitatively measure PRSV replication in inoculated papaya plants. PRSV accumulation increased dramatically in line 1280 by 12 DAI, but there was no evidence of PRSV in line 474 over the course of the experiment (Fig. 5).

**Figure 6:**
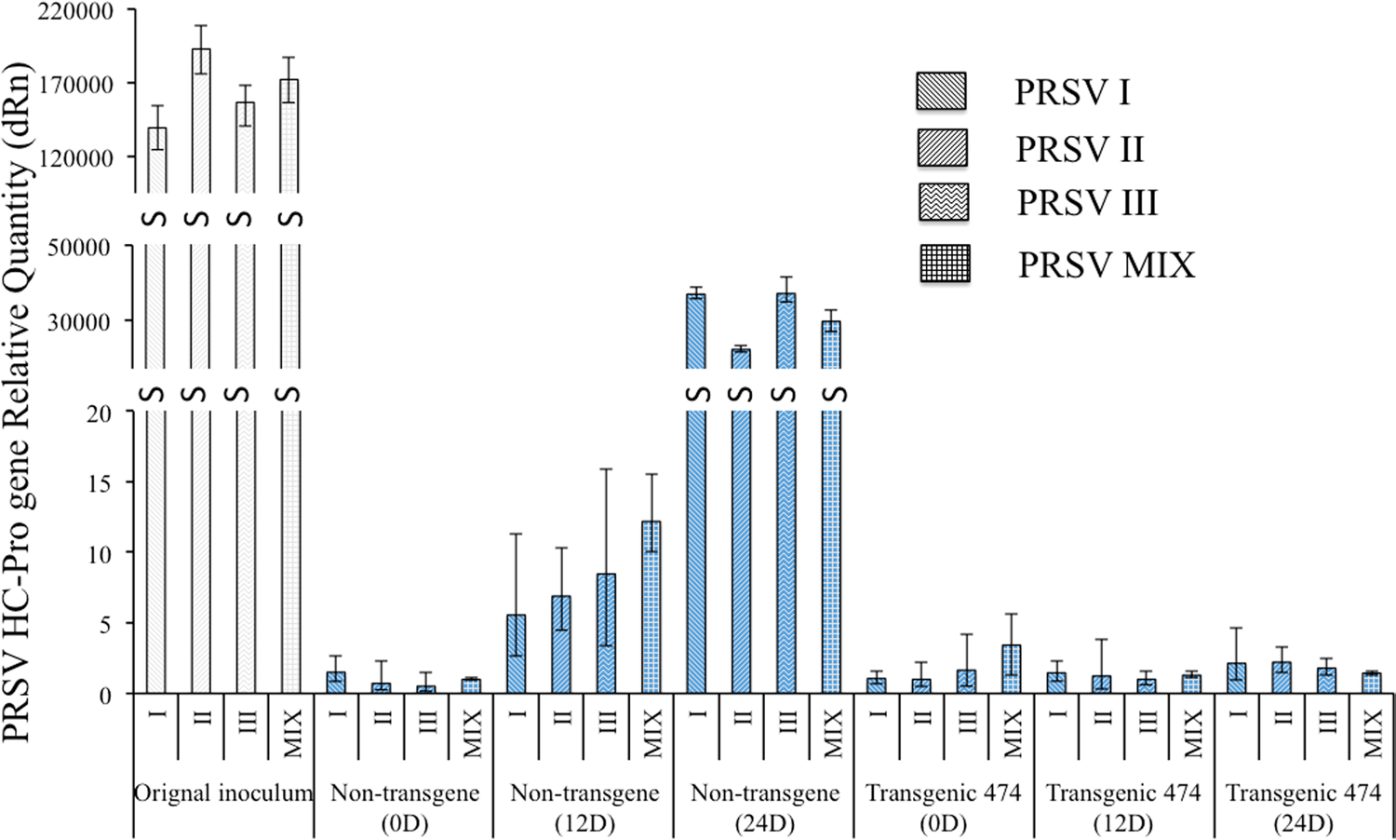
Quantitative real-time PCR was used to investigate the PRSV accumulation in transgenic and non-transgenic papaya leaves. Individual isolates F61d, F10d, and F21d represented PRSV Groups I, and II and III, respectively. PRSV MIX indicated the mixture of same amount of PRSV I, and II and III. The PRSV accumulation was measured at 0, 12 and 24 days after the inoculation.

The PRSV resistance of line 474 was also measured quantitatively by qPCR (Fig. 6). Virus accumulation within plants inoculated with individual isolates F61d, F10d, and F21d, representing PRSV subgroups I, II and III, respectively, as well as with a mixture of all three isolates, was investigated 0, 12, and 24 days after inoculation. Line 474 suppressed replication of all three PRSV isolates, whether applied individually or as a mixture,, while the non-transgenic line 1280 accumulated the PRSV isolates dramatically. Virulence of the three PRSV isolates, in terms of virus accumulation within the host, was not significantly different within either line 474 or line 1280.

### Northern blotting to detect accumulation of siRNA in transgenic papaya

**Figure 7:**
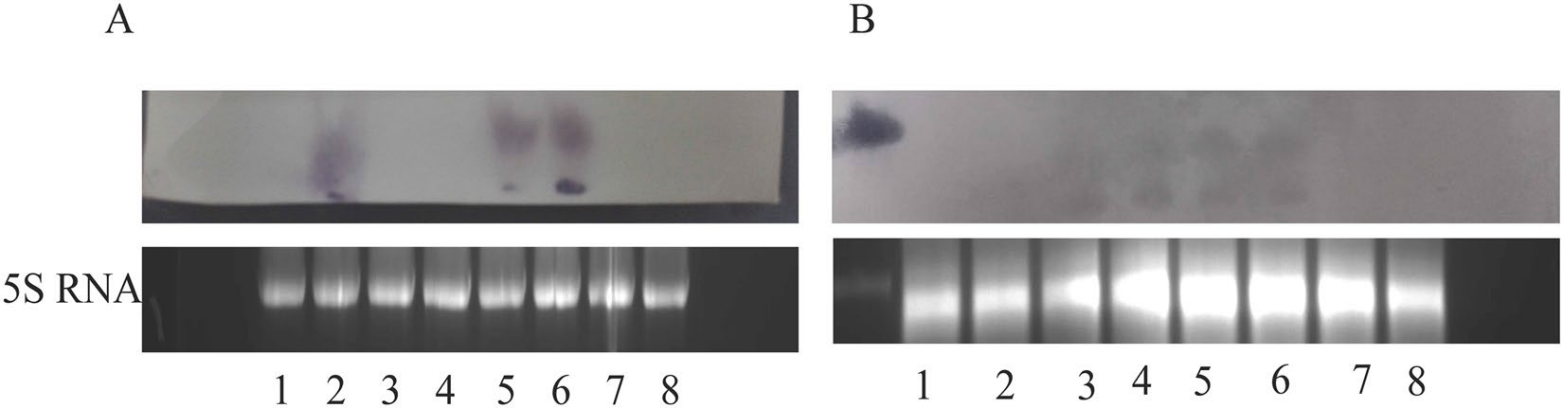
Northern blot analysis to investigate the siRNA accumulation on virus free papaya leaves (**A**) and virus challenged papaya leaves (**B**). The full-length gels are presented in Supplementary Figure 5 and Supplementary Figure 6. (**A**) Young tissue culture seedlings regenerated from embryogenic callus considered as virus free materials. Lane 1, 3 and 4 are non-transgenic line 1280. Lane 2, 5 and 6 are transgenic line 474. Lane 7 and 8 are traditional cultivars ‘ZhongBai’ and ‘Suizhonghong 48’ germinated from seeds as references. (**B**) PRSV challenged (24 days) papaya leaves were also estimated the siRNA accumulation. Lane 1 and 2 are transgenic lane 474 challenged with PRSV I isolate F61d. Lane 3 and 4 are transgenic lane 474 challenged with PRSV II isolate F10d. Lane 5 and 6 are transgenic lane 474 challenged with PRSV III isolate F21d. Lane 7 and 8 are pooled RNA mixture of non-transgenic line 1280 challenged with PRSV I, II and III respectively. Arrow indicated the probe self-hybridization dot (50 bp).

Evidence for PRSV resistance based on transgene silencing was strengthened by detection via Northern blotting of siRNAs in transgenic papaya seedlings (Fig. 7). Two siRNAs (<50 bp) were found in uninoculated transgenic papaya leaves. Our results also showed there were no corresponding siRNA products in non-trangenic line 1280 or traditional papaya cultivars ‘Zhongbai’ and ‘Suizhonghong 48’.

## Discussion

Breakdown of transgenic PRSV resistance, which depends on sequence homology between the transgene and attacking virus strain, is a major concern facing papaya cultivation, since genetically distinct strains of PRSV have been identified throughout the world^18^. In Hawaii, due to geographical isolation and the relative homology of PRSV strain existing there, the transgenic resistance to PRSV introduced in papaya cultivars Rainbow and SunUp in 1998 is still effective against local strains^8^. However, in areas like Hainan, China, where more diverse PRSV strains exist, a narrowly based transgenic resistance is expected to drive the emergence of more virulent virus strains and eventually succumb to the adapting virus population^16^. Consequently, papaya cultivars with resistance to a broad spectrum of PRSV strains are urgently needed by the papaya industry in Hainan.

Other researchers have suggested introducing the HC-Pro gene into transgenic lines to overcome the potential emergence of more virulent virus strains selected by transgenic crops currently using a CP target gene^11^. Our approach to achieving broad transgenic resistance to PRSV involved selecting a conserved region of the CP gene as the RNAi target sequence. Analysis of 53 PRSV isolates from Hainan revealed a 544-bp region of the CP gene that shared 97 to 100% identity among all isolates.

Particle bombardment has been commonly used as a gene transformation method^19–21^. Some researchers report that particle bombardment tends to produced multiple or damaged insertions^22^. However, the flexibility of the particle gun method allows control of the frequency of insertions and ability to produce either low- or high-copy events by altering the quantity of cassette DNA used in the bombardment^22^.

The use of PTGS to produce PRSV-resistant papaya is a proven approach to management of PRSV. We used an RNAi strategy to target a conserved region of the PRSV CP gene during virus replication to develop resistance to a broad spectrum of PRSV isolates from Hainan. Our results showed that transgenic line 474 produced two siRNAs with molecular weights of less than 50 bp. In short term (4-week) greenhouse experiments, transgenic line 474 showed resistance to PRSV strains that caused disease symptoms in non-transgenic controls, but evaluation of resistance durability must await longer term field trials using inoculated and uninoculated plants grown from seedling generations (Supplementary Figure 8).

## Methods

### Plant materials

Traditional papaya cultivars ‘Sunrise’, ‘Zhongbai’ and ‘Suizhonghong 48’ were collected in the lab. Genomic DNA from papaya cultivar ‘SunUp’ was provided by Hawaii Agriculture Research Center, Waipahu, HI. USA.

Embryogenic callus tissue from seeds of the papaya cultivar ‘Sunrise’ was cultured on the induction medium^23^. Papaya transformation and regeneration were carried out by particle bombardment as described previously^24^.

### PRSV isolates and inoculation bioassay

The PRSV isolates from Hainan province are divided into subgroup I, II and III^16^. Isolates F61d, F10d, and F21d from each subgroup, respectively, were maintained −80 °C. Virus inoculum was prepared by inoculating local papaya plants ‘Suizhonghong 48’ prior to each bioassay. Papaya leaf samples with the well-developed symptoms of PRSV were homogenized with phosphate buffer solution (PBS) 1:10 (w/v), followed by centrifugation at 6000 × g, 4 °C. The supernatant was used as inocula within 48 hours or stored at −80 °C.

Transgenic line and non-transgenic papaya plants with four to six leaves were used to evaluate plant resistance. The third or fourth leaf from the top was gently rubbed with quartz sand using a blade tip. A 10-μl aliquot of inoculum was applied to each rubbed spot. Inoculum stayed on the leaf surface 5 min prior to washing with distilled water. PBS buffer was used as a control inoculum. All inoculated plants were kept in a growth chamber with 16 h light at 28 °C and 8 h dark at 25 °C. The rate of infection from two independent tests, each with 12 plants per line, was statistically analyzed using Duncan’s multiple range tests. Individual isolates as well as a mixture of isolates F61d, F10d, and F21d were used to inoculate transgenic line and non-transgenic papaya line.

### Vector construction and plant transformation

The genetic variability among PRSV isolates in Hainan was investigated in a previous study^16^. In this study, in order to use hairpin construct to generate a higher efficiency of RNA silencing, we selected the conserved region of the CP genes of the different Hainan isolates as the RNAi target sequence (Supplementary Figure 1). Sequence analysis of the 544-bp region of the Hainan PRSV isolates shared 97 to 100% identity.

The conserved fragment of PRSV CP gene was used to design RNAi hairpin structures. The constructed CP hairpin structure was inserted into pCAMBIA2300-35S-OCS to construct the plant expression RNAi vector (Fig. 1).

The total RNA from papaya leaves infected with isolate F61d (PRSV I) was extracted with RNeasy Plant Mini Kit (QIAGEN). The first strand of cDNA was synthesized by using the ImProm-II Reverse Transcription System (Promega). The targeted CP fragment with restriction sites (Table 1) was amplified by high-fidelity DNA polymerase (NEB). Two “directional” CP fragments (CP sense strand was digested with Xho I and Bgl II, and CP reversed strand was digested with Sal I and BamH I) were ligated to pUCCRNAi plasmid^25^ to form the hairpin structure with a 201-bp intron. The hairpin structure was digested with Sal I and Pst I and inserted into pCAMBIA2300-35S-OCS to form the plant expression vector (Fig. 1 and Supplementary Figure 2)^26^.

Plasmid DNA was applied to 1.6 μm gold particles (Bio-Rad, Hercules, Calif.) that were inserted into embryogenic callus using a PDS 1000 Helium (Bio-Rad) particle bombardment system as previously described^24^. Each target plate, containing approximately 1 g fresh weight of embryogenic callus, was bombarded three times. The bombarded callus was placed on half-strength MS medium, pH 5.8, containing 10 mg/L 2, 4-dichlorophenoxyacetic acid, 3% sucrose, and 2.5% Phytagel for 10 days to recover, and then treated with 100 mg/L Kanamycin.

### PCR validation the transgenic lines

PCR was used to detect transformation events in plants regenerated from the callus lines. DNA was extracted from 100 mg of papaya leaves using the E.Z.N.A. High Performance (HP) Plant DNA Kit (Omega) according to the manufacturer’s instructions. We used target-gene-specific primers, CP1/CP2, and construct-specific primers, 35S-F/CP2, to detect inserted sequences, and endogenous papain gene primers, papainF/papainR, as a check for proper function of PCR assays (Table 1). PCR was performed on a thermocycler (Biometra) as follows: one cycle of initial denaturation at 94 °C for 3 min; 35 cycles of denaturation at 94 °C for 30 s, annealing at 58 °C for 30 s and extension at 72 °C for 30 s (60 s for the 35S-F/CP2 primers); and one cycle of final extension at 72 °C for 10 min.

Two primers, 474-61 F/474-61 R, were designed to amplify a 192-bp sequence of papaya DNA centered on the transgene insertion site of line 474. A nested PCR assay containing both the insert site-specific primers, 474-61 F/474-61 R, and transgene-specific primers, CP1/CP2, was designed to confirm transgenic lines. The amplification cycling program consisted of one cycle of initial denaturation at 94 °C for 3 min; 35 cycles of denaturation at 94 °C for 30 s, annealing at 58 °C for 30 s and extension at 72 °C for 2 min; and one cycle of final extension at 72 °C for 10 min.

The QX100^TM^ Droplet Digital PCR system (ddPCR, Bio-Rad, USA) was used with target-gene primers CP1/CP2 referenced to the endogenous papain gene to estimate the target-gene copy number in transgenic lines 474 and SunUp, following the manufacturer’s instructions. The ddPCR amplification cycling program began with an initial denaturation at 95 °C for 5 min, followed by 45 cycles of denaturation at 95 °C for 20 s, annealing and elongation at 58 °C for 30 s; and the final cycle of incubation at 98 °C for 10 min. QuantaSoft (V1.3.2.0) software was used to analyze the experimental data and quantify the PCR products. Three biological replicates per transgenic genotype x primer-set treatment, and three technical replicates per biological sample, were assayed.

### Southern blot analysis to confirm transgenic events

Papaya DNA was extracted with the E.Z.N.A. High Performance Plant DNA Maxi Kit (Omega) and used for Southern blotting. CP-gene probes were prepared by amplification with specific primers CP1 and CP2 (Table 1) and labeled with Digoxigenin-11-dUTP using DIG-High-Prime Kit (Roche). About 60 μg of genomic DNA was digested with Hind III and BamH I, separately. Electrophoresis of the genomic DNA was performed in 1.0% agarose gel. The DNA was transferred to nylon membranes (GE Healthcare) using the capillary siphon blot method^27^, and then fixed by UV-crosslinking (Scientz Biotechnology, LTD) at 243 nm for 45 min. Hybridization was performed overnight at 42 °C in a hybridization incubator (Model 2000 Robbins Scientific). Immunological detection was made with anti-Digoxigenin-AP following the product manual, and recorded with an EC3 imaging system (UVP, LLC).

### Northern blot analysis to investigate siRNA accumulation

Tissue cultured transgenic and non-transgenic papaya plants, virus free or 24 days after inoculation with PRSV, provided leaf RNA extracts via RNeasy Plant Mini Kit (QIAGEN) for investigation of siRNA accumulation. The integrity of RNA was checked with formaldehyde denaturing agarose gel electrophoresis^27^. The conserved 544-bp sequence of the PRSV CP gene was fragmented into 11 probes averaging 50 bp in length, of which probe CP544-10 (from 451 bp to 500 bp) and probe CP544-11 (from 501 bp to 544 bp) were later confirmed to produce efficient hybridization signals (Table 1). The Northern blot probes were labeled with Digoxigenin-11-dUTP using DIG-High-Prime Kit. Both 15% formaldehyde denaturing agarose gels (lab made) and 15% Mini-PROTEAN TBE-Urea Gels (Bio-Rad, USA) were used to separate the small RNAs by loading approximately 60-80 μg of RNA per sample, and transferring the separated bands to nylon membranes (GE Healthcare) using the capillary siphon blot method^27^. Hybridization was performed overnight at 42 °C in a hybridization incubator (Model 2000, Robbins Scientific). Immunological detection was the same as for the Southern blot, except that the containers, reagents and buffers were pre-treated with 0.1% DEPC (v:v) water to remove contaminating RNAase.

### High-efficiency thermal asymmetric interlaced PCR (*hi*TAIL-PCR)

The candidate transgenic lines were screened for insertion sites using the *hi*TAIL-PCR method^28^. The primers used are listed in Table 1. PCR products were cut from the agarose gel and purified with the E.Z.N.A. Gel Extraction Kit (Omega). The purified amplicons were cloned in a pMD18 vector (TaKaRa) following the manufacturer’s protocol, and then transformed into *Escherichia coli* DH5α competent cells. Three positive colonies from each transformation were sequenced with an ABI 3130xl Genetic Analyzer (Hitachi), and a result of identical sequences from all three colonies was considered confirmation of the actual sequence.

### RT-PCR and qPCR to detect PRSV-resistant lines

PRSV accumulation in leaves of transgenic and non-transgenic papaya plants was investigated after inoculation with individual PRSV isolates (F61d, F10d, or F21d), as well as a mixture of all three isolates. Each papaya genotype x PRSV isolate treatment had 5 biological replicates. The tissue cultured papaya plants were cultivated in the greenhouse and observed every four days for symptom development.

PRSV accumulation in papaya leaves after inoculation with the mixture of PRSV isolates was monitored in a time series of 0, 4, 8, 12, 16, 20, 24 and 28 days after inoculation (DAIs). For this assay, we used the PRSV HC-Pro gene as an indicator of the virus accumulation. Plant RNA was extracted with an RNeasy Plant Mini Kit (QIAGEN). First-strand cDNA was synthesized using the ImProm-II Reverse Transcription System (Promega) by starting with total RNA and oligo (dT)15. HC-Pro gene-specific primers were used for second-strand amplification and were able to amplify PRSV I, II and III (Table 1). The polymerase chain reaction (PCR) mixture contained 1 µl of cDNA, 0.5 µl of each primer (10 µM), 2 µl of 10 × PCR buffer, 1 µl of dNTP, 0.25 U of Taq DNA polymerase (Takara), and nuclease-free water to a final volume of 20 µl. The amplification consisted of 35 cycles at 94 °C (30 s), 50 °C (1 min), and 72 °C (1 min); the initial cycle incorporated a melting step of 94 °C for 3 min and the final cycle a synthesis step of 72 °C for 10 min. PCR products were electrophoresed on 1.5% agarose gel, and visualized after ethidium bromide staining.

Quantitative real-time PCR investigated the PRSV accumulation in papaya leaves at 0, 12 and 24 days after the inoculation with individual isolates (F61d, F10d, and F21d) and PRSV mix. A Mx3005 P Thermocycler (Strata Gene) was used in the amplification. The accumulation of PRSV was detected by SYBR Premix Ex Taq (TaKaRa) with signal correction using ROX reference dye.

## Electronic supplementary material

Supplementary Information
