# Publisher Correction: Use of RNAi technology to develop a PRSV-resistant transgenic papaya

**DOI:** 10.1038/s41598-017-16198-4

**Published:** 2017-11-22

**Authors:** Ruizong Jia, Hui Zhao, Jing Huang, Hua Kong, Yuliang Zhang, Jingyuan Guo, Qixing Huang, Yunling Guo, Qing Wei, Jiao Zuo, Yun J. Zhu, Ming Peng, Anping Guo

**Affiliations:** 1 Institute of Tropical Bioscience and Biotechnology, Chinese Academy of Tropical Agriculture Sciences, 571101 Haikou Hainan, China; 2 Hawaii Agriculture Research Center, 96797 Waipahu HI, USA; 3 School of Basic and Life Science, Hainan Medical University, Haikou 571199 Hainan, China; 4 Institute of Banana and Plantain, Haikou Substation, Chinese Academy of Tropical Agriculture Sciences, 570102 Haikou Hainan, China

*Scientific Reports* **7**:12636; doi:10.1038/s41598-017-13049-0; Article published online 03 October 2017

The original HTML version of this Article contained errors in Table 1, where text was inadvertently inserted on three occasions.

For the Target gene ‘CP hairpin sense strand’ with Primer name ‘CP-RNAi-P2’, the Primer sequence (5′-3′) incorrectly read “GAcomment=” Underline the selected text “AGATCTYCACGAGCCCTATCAGGTGTCTTT Bgl II” and now reads “GAAGATCTYCACGAGCCCTATCAGGTGTCTTT Bgl II”.

For the Target gene ‘CP hairpin reverse strand’ with Primer name ‘CP-RNAi-P3’, the Primer sequence (5′-3′) incorrectly read “GCcomment=” Underline the selected text “comment=” Underline the selected text “GTCGACTCAACGCCGGAACTAGTGGAACTT Sal I” and now reads “GCGTCGACTCAACGCCGGAACTAGTGGAACTT Sal I”.

For the Target gene ‘CP hairpin reverse strand’ with Primer name ‘CP-RNAi-P4’, the Primer sequence (5′-3′) incorrectly read “CGcomment =” Underline the selected text “comment =” the selected text“comment =” Underline the selected text “GGATCCTCACGAGCCCTATCAGGTGTCTTT BamH I” and now reads “CGGGATCCTCACGAGCCCTATCAGGTGTCTTT BamH I”.

These errors have been corrected in the HTML version of the Article; the PDF version was correct from the time of publication.

