# Supplementary material for: Use of RNAi technology to develop a PRSV-resistant transgenic papaya
Source: Sci Rep. 2017 Oct 3;7:12636. doi: 10.1038/s41598-017-13049-0 (PMC5626737; doi:10.1038/s41598-017-13049-0)
Supplement: Supplementary file 1 — Supplementary Information [file 41598_2017_13049_MOESM1_ESM.pdf]

Use of RNAi technology to develop a PRSV-resistant transgenic papaya

Ruizong Jia [1, 2, a], Hui Zhao [1, a], Jing Huang [1, 3, a], Hua Kong [1], Yuliang Zhang[1], Jingyuan Guo [1], Qixing Huang [1], Yunling Guo [1], Qing Wei [1, 4], Jiao Zuo[1], Yun J. Zhu[1, 2, *], Ming Peng [1, *], & Anping Guo [1, *]

Supplementary Info

Supplementary Figure 1
Supplementary Figure 2
Supplementary Figure 3
Supplementary Figure 4
Supplementary Figure 5
Supplementary Figure 6
Supplementary Figure 7

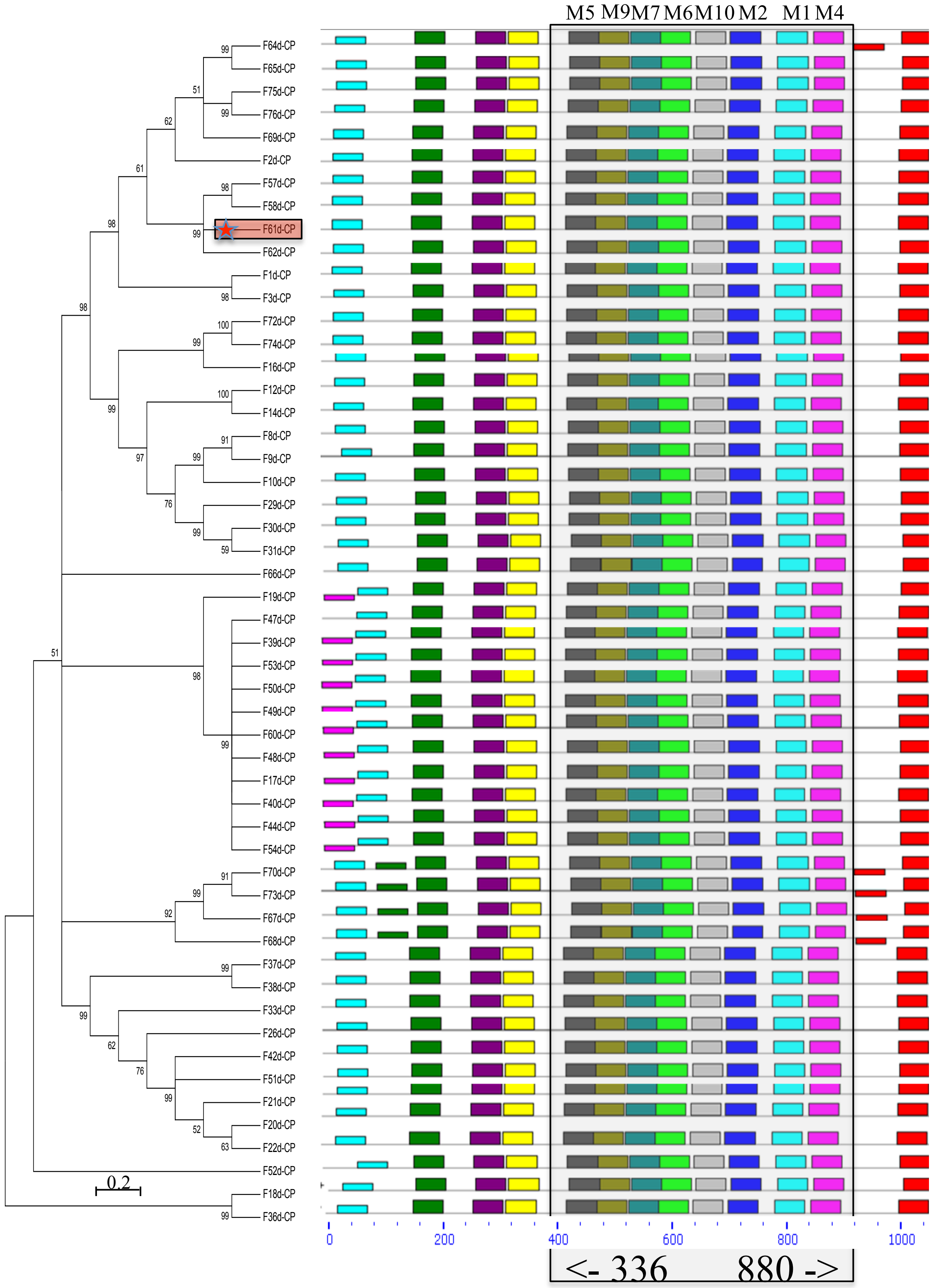

Supplementary Figure 1. A neighbor-joining phylogenetic tree of 52 CP gene

sequences (GenBank accession numbers KF002591 to KF002709) from Hainan isolates of PRSV (F-1d CP to F-76d CP) is shown on the left (Zhao et al., 2016). The block schematics of the CP gene to the right of the tree show different motifs in different colors. Sequence analysis of the CP genes with MEME (v. 4.11.2) revealed a mostly conserved 544-bp region (indicated by the shaded frame) containing highly homologous motifs. . The conserved region of PRSV isolate F61d, indicated with a star (CP gene, Acc# KF002696), was selected as the PCR template for our transformation construct, since it was the most identical to the conserved regions of all other CP sequences (97 to 100% homology) Bricks in same color represent the same motifs. M1 indicates motif 1, its regular expression is:

GGACGGCAGTGTCAGTAACAAGGAAGAAAACACGGAGAGACACACAGTGG.

M2 indicates motif 2, its regular expression is:

ACACCTGATAGGGCTCGTGAAGCTCA[TC]ATGCAGATGAA[GA]GCTGCAGCGCT.

M4 indicates motif 4, its regular expression is:

GAGACATGCACTCTCTCCTGGGTATGCGCAATT[AG]AATACTCGC[GA]CTAGT;

M5 indicates motif 5, its regular expression is:

AACACTCG[TC]GCCACTCAATCTCAATTTGA[AG]AAGTGGTATGAGGGAGTGAG

M6 indicates motif 6, its regular expression is:

GCATA[TC]ATCGC[GA]AAGAG[GA]AATGCAACTGAGA[GA]GTACATGCCGCGGTATGG

M7 indicates motif 7, its regular expression is:

GCAACTCCTTCATT[TC]AGGCA[AG]AT[CT]ATGGCTCACTTCAGTAACGCGGCAGA

M9 indicates motif 9, its regular expression is:

GAGAGAGATAG[AG]GATGTCAA[TC]GCCGGAACTAGTGGAAC[TC]TTCACTGTTCC

M10 indicates motif 10, its regular expression is:

AATTTGACTGACATTAGTCTCGC[TC][CA]GATATGCTTTCGATTTCTATGAGGT

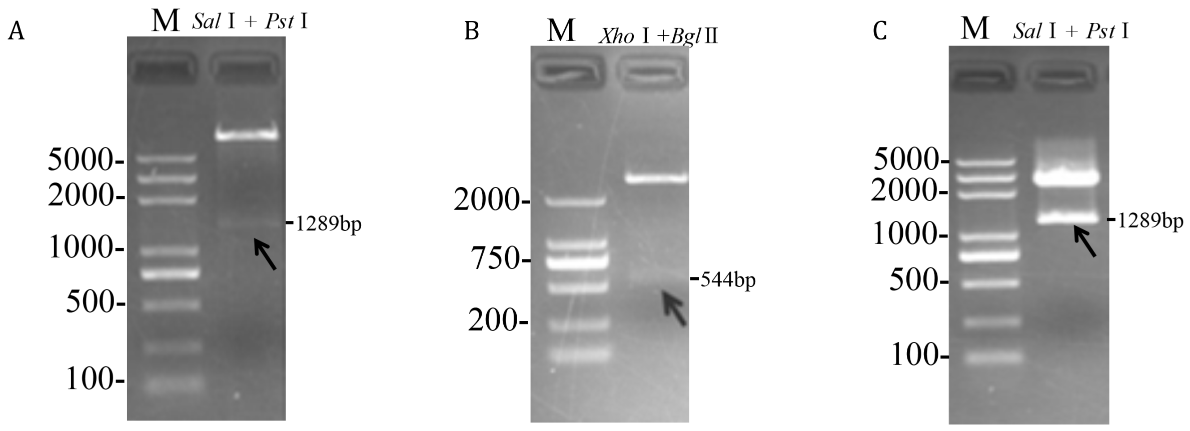

Supplementary Figure 2. RNAi vector validated by restriction enzyme digestion. Arrows indicated expected double enzyme digestion products, respectively. M indicated the DNA molecular weight (bp). (A) Sal I and Pst I double digested pCAMBIA2300-35S-OCS plasmid, a 1289 bp fragments contains sensed and reversed 544 bp fragment and 201 bp intron. (B) CP sense strand was digested with Xho I and Bgl II, and CP reversed strand was digested with Sal I and BamH I (not show). (C) Sal I and Pst I double digested pUCCRNAi plasmid 25 to form the hairpin structure with a 201-bp intron.

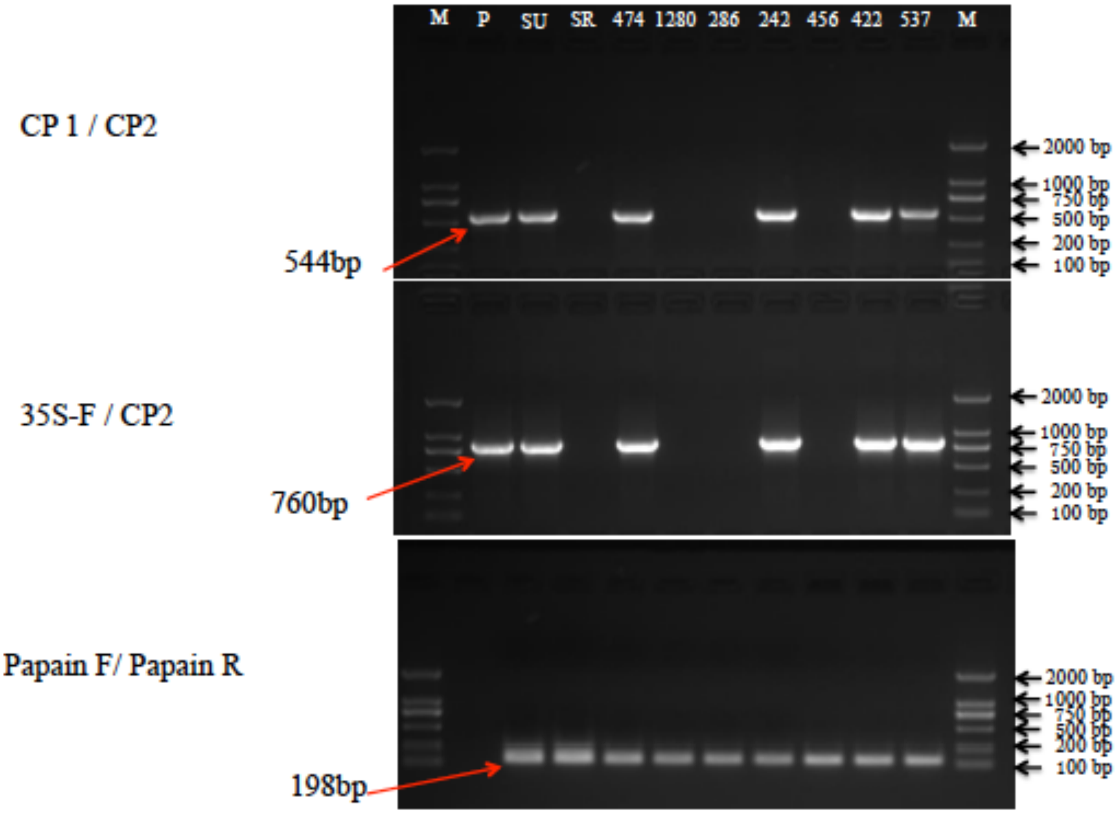

Supplementary Figure 3. PCR validation of the transgenic event in difference lines. Left panel of agarose gel images indicated difference primers combination: the CP1/CP2 is the targeted CP gene primer pair. 35S-F/CP2 is the vector-specific primer pair contains partial of 35S promoter and partial CP gene. Papain F/ Papain R is papaya reference gene primers. M: indicated the DNA molecular weight, P indicates plasmid DNA, SU indicated 'SunUp' DNA, SR indicated 'SunRise' DNA before transformation. The rest of numbers are candidate transgene lines. Arrows on the right panel indicated the expected PCR products.

A

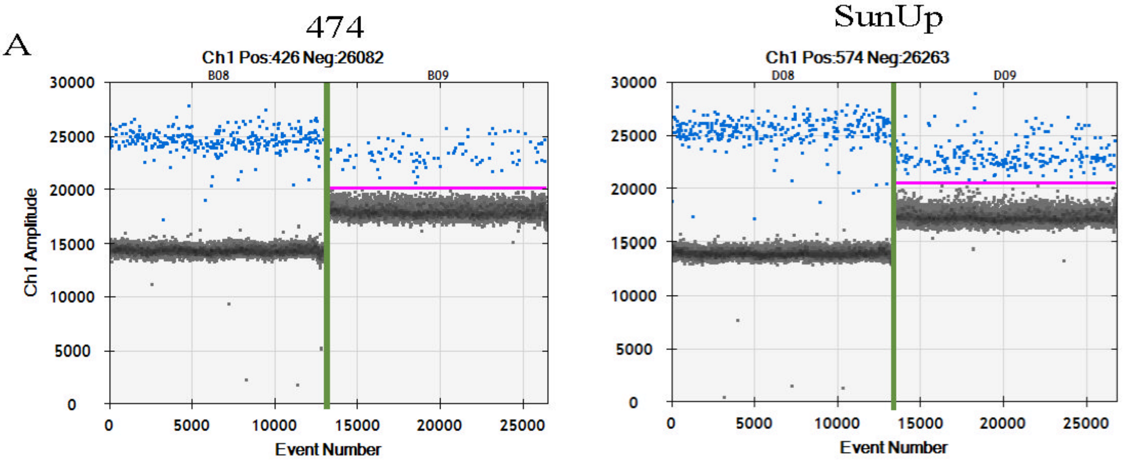

B

Sample	Gene	Concentration (copies/μL)	Copy number	Ratio
474	Papain	29.2/29.7/30.1	-	0.3
	CP	8.8/9.0/8.5	1	
SunUp	Papain	29.1/29.5/28.7	-	0.7
	CP	21.7/19.9/20.5	2.4	

Supplementary Figure 4. Droplet Digital PCR method estimating the number of gene copies inserted into the plant genome. (A) Amplification scatter charts of each droplet in transgenic line 474 and the positive control 'SunUp'. (B) The calculated insert gene copy number for 474 and 'SunUp'. Each dot represents droplet amplification fluorescence signals: black dots represent no template detected in the droplets and no amplification reaction; blue dots represent positive amplification droplets; the pink line is the threshold of positive droplets (>20,000). Dots on the left of green line represent amplification of the internal reference gene (papain); dots on the right of the green line are the amplification of targeted CP fragments

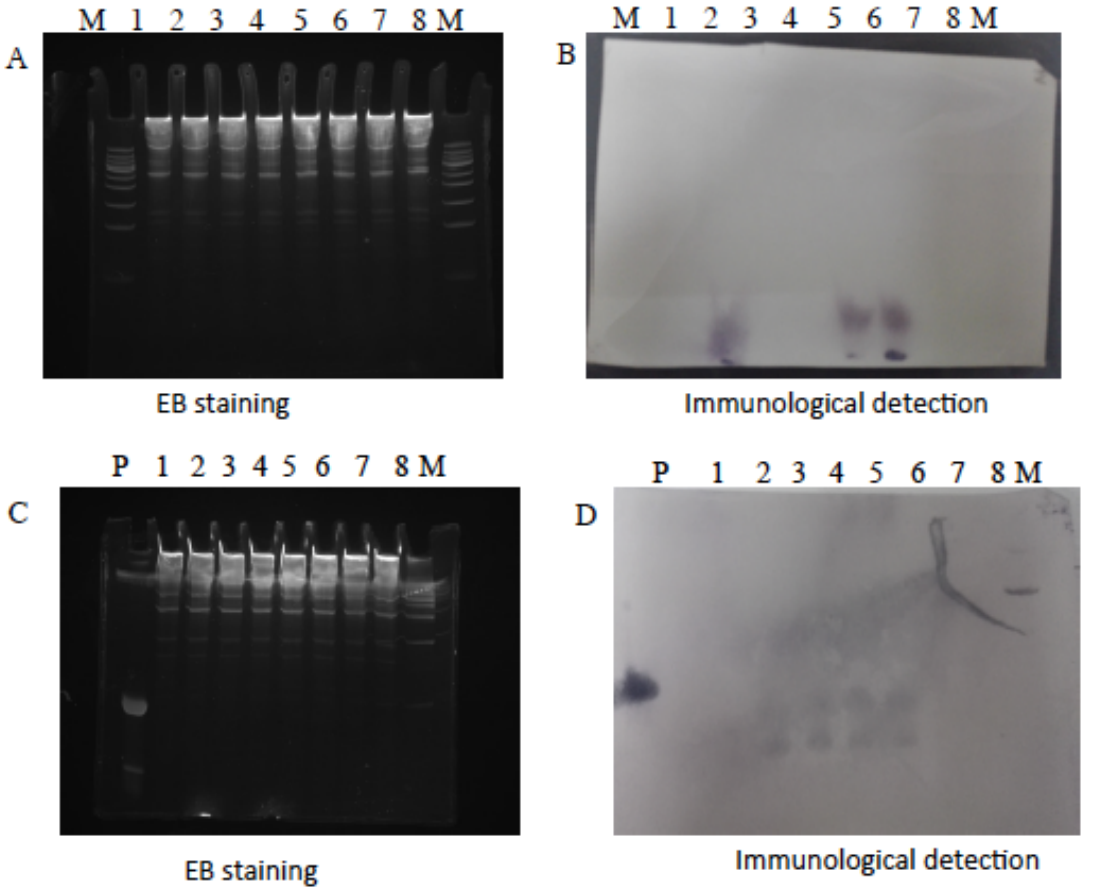

Supplementary Figure 5. Northern blot analysis to investigate the siRNA accumulation on virus free papaya leaves (EB staining image A and Immunological detection image B) and virus challenged papaya leaves (EB staining image C and Immunological detection image D). The full-length gels are presented in Supplementary Figure 2 and Supplementary Figure 3. A: Young tissue culture seedlings regenerated from embryogenic callus considered as virus free materials. Lane 1, 3 and 4 are non-transgenic line 1280. Lane 2, 5 and 6 are transgenic line 474. Lane 7 and 8 are traditional cultivars 'ZhongBai' and 'Suizhonghong 48' germinated from seeds as references. B: PRSV challenged (24 days) papaya leaves were also estimated the siRNA accumulation. Lane 1 and 2 are transgenic lane 474 challenged with PRSV I isolate F61d. Lane 3 and 4 are transgenic lane 474 challenged with PRSV II isolate F10d. Lane 5 and 6 are transgenic lane 474 challenged with PRSV III isolate F21d. Lane 7 and 8 are pooled RNA mixture of non-transgenic line 1280 challenged with PRSV I, II and III respectively. Arrow indicated the probe self-hybridization dot (50 bp). M indicated DNA molecular marker (50bp ladder) without denture at 95 ^{o}C prior to loading. P indicated the probe oligo.

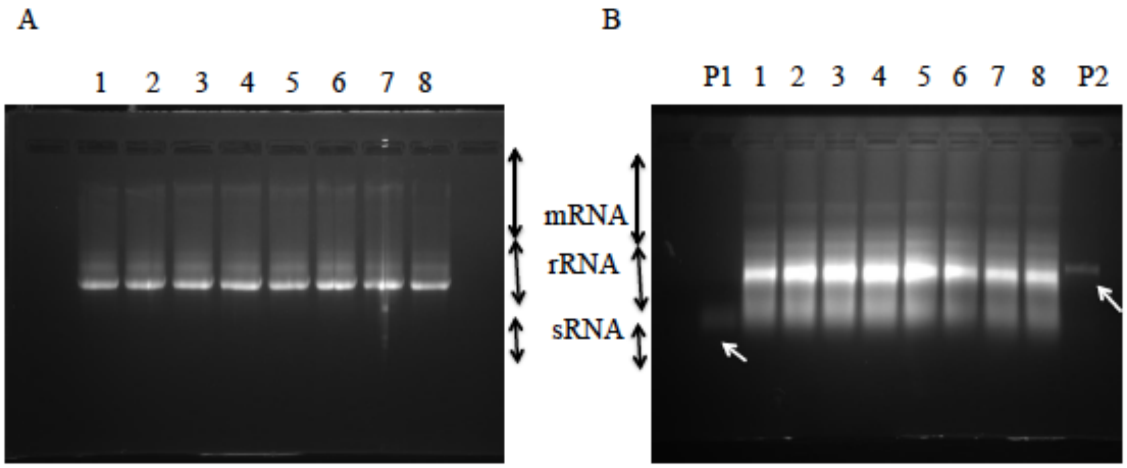

Supplementary Figure 6. The full-length formaldehyde denaturing agarose gel electrophoresis to check he integrity of RNA. A: Young tissue culture seedlings regenerated from embryogenic callus considered as virus free materials. Lane 1, 3 and 4 are non-transgenic line 1280. Lane 2, 5 and 6 are transgenic line 474. Lane 7 and 8 are traditional cultivars 'ZhongBai' and 'Suizhonghong 48' germinated from seeds as references. B: PRSV challenged (24 days) papaya leaves were also estimated the siRNA accumulation. Lane 1 and 2 are transgenic lane 474 challenged with PRSV I isolate F61d. Lane 3 and 4 are transgenic lane 474 challenged with PRSV II isolate F10d. Lane 5 and 6 are transgenic lane 474 challenged with PRSV III isolate F21d. Lane 7 and 8 are pooled RNA mixture of non-transgenic line 1280 challenged with PRSV I, II and III respectively. Arrow in lane P1 indicated the probe oligo. Arrow in lane P2 indicated the 544bp CP PCR products.

A B

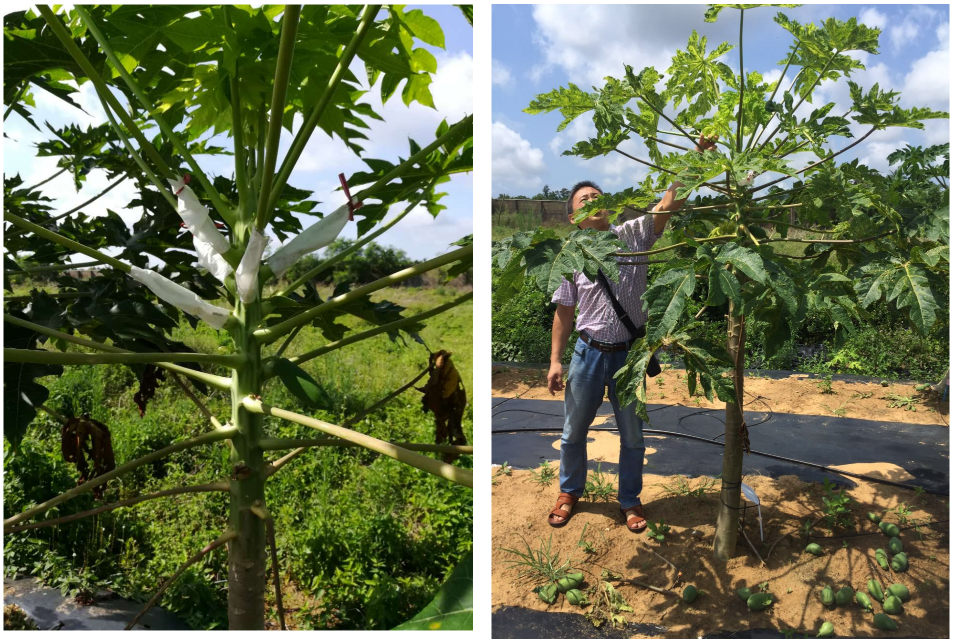

Supplementary Figure 7. Field trail of transgenic line 474 (A) with no PRSV symptoms and non transgenic line 1280 (B) were severely infected by PRSV. All the plants were planted in open field with out inoculation.
